# Scaling of next generation solution processed organic and perovskite solar cells

**DOI:** 10.1038/s41467-018-05514-9

**Published:** 2018-12-10

**Authors:** Paul Meredith, Ardalan Armin

**Affiliations:** Department of Physics, Swansea University, Singleton Park, Swansea, SA2 8PP Wales UK

## Abstract

Why, despite considerable R&D efforts and significant translational investment over the past 20 years, has the technology of solution-processed thin film solar cells not become a commercial reality? The manufacturing cost-to-power conversion efficiency ratio seems persuasive, as do the energy payback and embodied energy metrics. As new perovskite-based semiconductors achieve impressive efficiencies and organic semiconductors enjoy a resurgence, the lab-to-manufacturing translation and scaling questions require urgent attention. This comment addresses the challenges in solution processable photovoltaic technologies faced by scientists and engineers in addressing these questions, and highlights the concept of thick junctions as a promising solution.

## Next-generation solution-processed thin film PV

The drive towards ever lower cost solar energy continues to motivate intense activity in next-generation photovoltaics (PVs). Semiconductors which can be solution processed are one avenue of prolific R&D since they genuinely offer the prospect of ultra-low-$/watt manufacturing with reduced embodied energy. Organic semiconductors and organohalide perovskites are two such systems. Organic solar cells (OSCs) containing n-type and p-type polymers and small molecules have reached power conversion efficiencies (PCEs) of >13% at the laboratory scale^1,2^. Perovskite solar cells (PSCs) either as planar junctions or mesoporous scaffolds now exceed 22% in a remarkably short development period of <5 years^3^. Both technologies, although based upon very different semiconductors, share common architectures, namely a thin junction sandwiched between work function-modified charge-selective contacts, one of which must be transparent and conducting (the transparent conducting electrode or TCE). In the world of OSCs and PSCs ‘thin’ means 100–300 nm: a challenging thickness regime for high-throughput, high-yield, low-cost manufacturing of large area solution-processed optoelectronics. The definition of these challenges and their solutions, particularly the ‘thick junction’ concept (>500 nm), are the subjects of this Comment.

## The scaling issue: simple parameterization

**Fig. 1:**
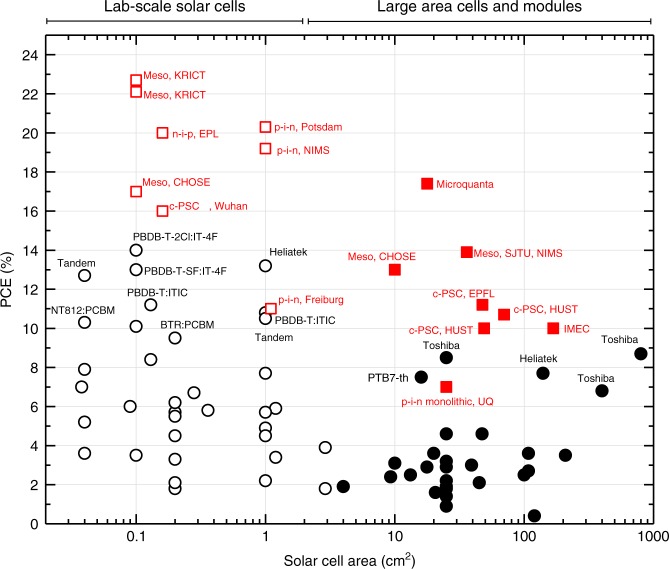
OSC and PSC power conversion efficiencies for lab-scale and large area cells and modules. Open red squares, lab-scale PSC; filled red squares, large area and module PSC; open black circles, lab-scale OSC; filled black circles, large area and module OSC. c-PSC carbon stack PSC; material systems, reporting institutions partially indicated alongside record references: PBDB-T-2CI:T-4F^1,2;^ Meso, KRICT^3;^ p-i-n monolithic UQ^7;^ NT812-PCBM^11;^ c-PSC, EPFL^14;^ p-i-n-Potsdam^15;^ Meso, SJTU, NIMS^16;^ Meso CHOSE^17^

**Fig. 2:**
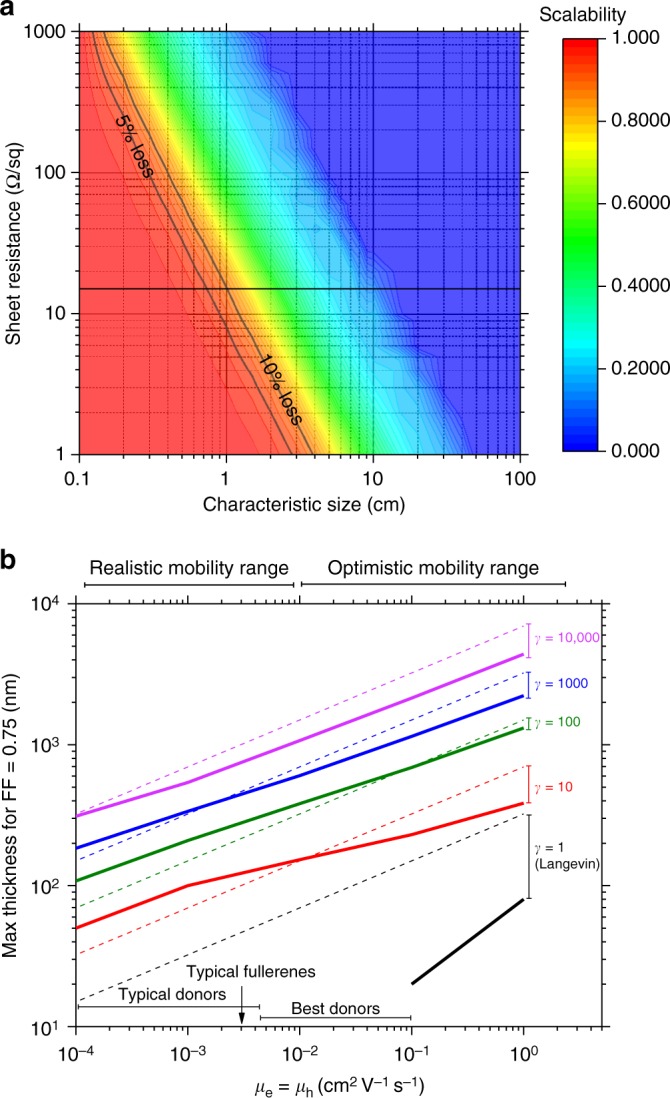
Scalability and maximum junction thickness versus critical transport parameters. **a** Scalability as a function of the TCE sheet resistance and the characteristic size (active area, strip width or grid pitch) for a hypothetical solar cell with short circuit current density of 20 mA/cm^2^ typical of a high-efficiency OSC or PSC. **b** Maximum acceptable junction thickness to deliver a 0.75 FF as a function of the carrier mobility *μ* (for balanced electron and hole mobilities) plotted for different bimolecular recombination reduction factors (*γ*). The numerical calculations were performed for an active layer with a bandgap of 1.6 eV under AM1.5 G illumination. Dashed lines represent predictions from the Neher analytical model^12^ and the solid lines a more complete finite element simulations

The aforementioned record efficiencies have all been achieved on small area devices («1 cm^2^). In general, these high PCEs do not scale, that is, they do not translate to sizes which are meaningful. This is clearly demonstrated by Fig. 1 which shows published PCEs for lab-scale and large area cells and modules. The absence of data points in the top right-hand quadrant is stark, although perovskite junctions have begun populating this space. The reasons for the scaling issue are multi-faceted: some are well appreciated but others only just emerging. Ultimately, in the limit that the series resistance is much smaller than the shunt, the current–voltage (*I–V*) characteristics of any solar cell can be parametrized by the Shockley equation^4:^

1 $$I = I_0\left\{ {{\mathrm{exp}}\left[ {\frac{{q\left( {V - IR_{\mathrm{s}}^{{\mathrm{tot}}}} \right)}}{{n_{{\mathrm{id}}}k_{\mathrm{B}}T}}} \right] - 1} \right\} - I_{\mathrm{L}} + \frac{{V - IR_{\mathrm{s}}^{{\mathrm{tot}}}}}{{R_{{\mathrm{sh}}}^{{\mathrm{tot}}}}},$$

where the series and shunt resistances are, respectively: $R_{\mathrm{s}}^{{\mathrm{tot}}} = R_{\mathrm{s}} + \tilde R_{{\mathrm{elec}}}$ and $\frac{1}{{R_{{\mathrm{sh}}}^{{\mathrm{tot}}}}} = \frac{1}{{R_{{\mathrm{sh}}}}} + \mathop {\sum }\nolimits_j \frac{1}{{r_{D_j}}}$. Also, *I* is the total current composed of the light (*I*_L_), dark and shunt leakage components. All other parameters have their usual definitions, namely elementary charge (*q*), ideality factor (*n*_id_), Boltzmann’s constant (*k*_B_) and absolute temperature (*T*). This simple equation, although empirical, reveals the importance of the series and shunt resistances that characterize the diode and which have several components extrinsic and intrinsic to the active layer. For example, $\tilde R_{{\mathrm{elec}}}$ is the average resistance of the electrode; this resistance is distributed on the TCE as the sheet resistance; and $r_{D_j}$ are the resistances of point defects in the active layer resulting in leakage. This simplistic view is insightful and leads to an understanding of why scaling has been such a challenge in OSCs and PSCs which can be summarized as follows:

Firstly, sheet resistances of commercial TCEs are ~10–20 Ω/sq. At these sheet resistances, modelling of the *I–V* distribution shows that electrode collection path lengths >1 cm cause significant power loss or fill factor (FF) reduction which we quantify according to the scalability (defined as the ratio of the small-cell to large-cell performance in Fig. 2a). Hence, while lab-scale devices often made using non-scalable spin coating may yield high PCEs, larger cells do not and almost exclusively OSC and PSC small modules are composed of serially interconnected narrow strips to mitigate the effect.

Secondly, point defects in the active layer reduce the total shunt resistance and again result in power loss. In thin junction solar cells where the active layer may be ~100 nm thick, the probability of being affected by the defects scales exponentially with area. This is particularly troublesome for solution-processed cells made using fast throughput printing techniques (i.e. not spin coating)—they are plagued by low yield due to high defect densities. Again, the solution to date has been to minimize the defect impact by implementing narrow strip architectures.

Finally, a solution-processed thin junction has typical thickness non-uniformity of order 10s of nm dependent upon material. Planar PSCs composed of polycrystalline grains of perovskite may be even rougher. The thinner the active layer, the more serious the impact of thickness inhomogeneities, resulting in distributed carrier generation profiles, series and shunt resistances. The question is how do we mitigate these effects, and, moreover, enable low-cost, high-throughput manufacturing techniques capable of near-perfect yield over large areas?

## The scaling issue: possible solutions and thick junctions

Figure 2a shows that the TCE sheet resistance must be reduced to <1 Ω/sq to tolerate strip widths >5 cm, that is, approaching the dimensions of a typical c-Si cell. This would dramatically reduce manufacturing complexity, deliver additional flexibility for current–voltage engineering and potentially make thin film solar cells compatible with c-Si for tandems. However, that nirvana is distant, and we have no TCEs that are sufficiently transparent with such low sheet resistances: state-of-the-art is ~8 Ω/sq with indium tin oxide, fluorine-doped tin oxide or combinations of nanowires^5^. Another potentially more generic strategy is to mimic c-Si cells and create metallic grids on the TCE. It is not yet clear what the optimal configuration for such an approach is, but OSC and PSC large area monolithic devices of active area 5 cm × 5 cm have been reported using rudimentary metallic line grids with pitch ~1 cm^6,7^, which maintain FFs versus the lab-scale equivalents. The challenge in coating a thin junction over >500 nm high metallic lines is significant and boils down to creating conformal, defect-free active layers: coating solution viscosity and wetting combine with crystallization and morphology engineering to create a multi-parameter processing problem. The question of the active layer thickness is thus critical: moving to the thick junction regime where the active layer exceeds 500 nm may be key to unlocking the metallic grid strategy.

The thick junction concept is also gaining momentum in the context of mitigating defect and inhomogeneity issues. This is particularly so for OSCs where traditionally the highest efficiency organic semiconductor blends only function in the thin junction limit (~100 nm) due to transport limitations—notably the relative efficiencies with which carriers are extracted after photogeneration in competition with recombination^8,9^. In the transport regime occupied by organic semiconductors (mobilities <0.1 cm^2^/Vs) imbalanced transport leads to increased bimolecular recombination (i.e. non-geminate free carrier recombination) and associated FF reduction and power loss^10^. This effect has restricted junction thicknesses to <200 nm. However, new architectural and materials-level innovations specifically targeting more balanced transport are delivering impressive thick junction cells, for example: BTR-PCBM (benzodithiophene terthiophene rhodanine: [6,6]-phenyl-C71-butyric acid methyl ester), which maintains a PCE >9% at 300 nm junction thickness; NT812-PCBM (poly-naphtho[1,2-*c*:5,6-*c′*]bis[1,2,5]thiadiazole), which maintains a PCE >8% out to 1000 nm^10,11^. In both cases, the bimolecular recombination rate is suppressed by >100 relative to the Langevin rate (~800 for NT812)—this suppression metric is termed the reduction factor *γ*. The next step for such systems will be to scale them, potentially onto a grid, and examine the extent to which this structure–property relationship holds. A useful extension to this thinking has recently been developed by Neher et al.^12^ who derived a modified Shockley equation to predict the FF and maximum junction thickness. This analysis has several in-built assumptions such as perfect contacts, no space-charge effects and uniform optical generation profile, but is parameterized in terms of the carrier mobilities and *γ*. A similar but numerical analysis which incorporates space charge effects and optical profile is presented in Fig. 2b for a hypothetical cell with ohmic contacts and balanced electron and hole mobilities. Both approaches predict maximum affordable thicknesses for recombination-free charge collection (FF >0.75) of order 100s of nm when *γ* is ~1000 and carrier mobilities >10^−3^ cm^2^/Vs. These analyses provide basic design rules for how to deliver non-transport-limited thick junctions, particularly using OSC combinations.

It is also important to consider whether the above considerations also limit PSC junction thicknesses. In this regard, relatively balanced (and high) mobilities observed in both mesoporous scaffold and planar perovskite cells mean that they are not transport limited^13^. Particularly in the planar architecture, the junction thickness is morphologically constrained due to the emergence of shunt defects arising from large polycrystalline grains. Mesoporous scaffolds deliver junctions >500 nm without the perovskite crystallinity having negative shunt impacts. The so-called carbon stack cells containing perovskite infused mesoporous TiO_2_ and ZrO_2_ layers of thickness approaching a micron are a demonstration of the tolerance of PSCs to bimolecular recombination^14^. In fact, the carbon stack cell is a manifestation of all the concepts discussed in this Comment: it presents an architecture which is potentially scalable and tolerant to processing variances, and while not yet optimized, is amenable to simple low-cost printing methods. Combining the carbon stack PSC with an appropriate TCE metallic grid could deliver the first scaled, solution-processed thin film PV platform.

To conclude, we have outlined the challenges inherent in scaling solution processable thin film solar cells. We focus on the two cases of OSCs and PSCs, but the concepts discussed are pertinent to other technologies such as inorganic nanocrystals. An emerging solution to several of the key issues is the creation of efficient thick junctions (>500 nm) with balanced carrier transport and amenable to low-cost, large area printing techniques. This would dramatically improve manufacturing viability, as well as allowing the use of back contact grids to mitigate TCE limitations. Scientists and engineers are working together to deliver 15% efficient solution-processed photovoltaic modules from 20% lab cells.

## References

[1] Zhang Shaoqing, Qin Yunpeng, Zhu Jie, Hou Jianhui (2018). Over 14% Efficiency in Polymer Solar Cells Enabled by a Chlorinated Polymer Donor. Advanced Materials.

[2] Zhao W (2017). Molecular optimization enables over 13% efficiency in organic solar cells. J. Am. Chem. Soc..

[3] Yang WS (2017). Iodide management in formamidinium-lead-halide-based perovskite layers for efficient solar cells. Science.

[4] Armin, A. & Meredith, P. in *World Scientific handbook of Organic Optoelectronics*, Vol. 2 Organic Photovoltaics (eds So, F. & Thompson, B.) Ch. 7 (World Scientific Publishing, New York, 2018).

[5] Elmer K (2012). Past achievements and future challenges in the development of optically transparent electrodes. Nat. Photon..

[6] Armin A (2015). Efficient, large area, and thick junction polymer solar cells with balanced mobilities and low defect densities. Adv. Energy Mater..

[7] Hambsch M, Lin Q, Armin A, Burn PL, Meredith P (2016). Efficient, monolithic large area organohalide perovskite solar cell. J. Mater. Chem. A.

[8] Bartesaghi D (2015). Competition between recombination and extraction of free charges determines the fill factor of organic solar cells. Nat. Commun..

[9] Kaienburg P, Rau U, Kirchartz T (2016). Extracting information about the electronic quality of organic solar-cell absorbers from fill factor and thickness. Phys. Rev. Appl..

[10] Armin A (2016). Reduced recombination in molecular nematic liquid crystalline: fullerene solar cells. Adv. Energy Mater..

[11] Jin Y (2016). A novel naphtho[1,2-*c*:5,6-*c*′]Bis([1,2,5]thiadiazole)-based narrow-bandgap π-conjugated polymer with power conversion efficiency over 10%. Adv. Mater..

[12] Neher D, Kniepert J, Elimelech A, Koster LJA (2016). A new figure of merit for organic solar cells with transport-limited photocurrents. Sci. Rep..

[13] Herz LM (2017). Charge-carrier mobilities in metal halide perovskites: fundamental mechanisms and limits. ACS Energy Lett..

[14] Grancini G (2017). One-year stable perovskite solar cells by 2D/3D interface engineering. Nat. Commun..

[15] Stolterfoht Martin, Wolff Christian M., Márquez José A., Zhang Shanshan, Hages Charles J., Rothhardt Daniel, Albrecht Steve, Burn Paul L., Meredith Paul, Unold Thomas, Neher Dieter (2018). Visualization and suppression of interfacial recombination for high-efficiency large-area pin perovskite solar cells. Nature Energy.

[16] Chen H (2017). A solvent- and vacuum-free route to large-area perovskite films for efficient solar modules. Nature.

[17] Yaghoobi N, Zendehdel M, Cina L, Matteocci F, Di Carlo A (2018). A crystal engineering approach for scalable perovskite solar cells and module fabrication: a full out of glove box procedure. J. Mater. Chem. A.

